# Green Tea Extracts Attenuate Brain Dysfunction in High-Fat-Diet-Fed SAMP8 Mice

**DOI:** 10.3390/nu11040821

**Published:** 2019-04-11

**Authors:** Shintaro Onishi, Shinichi Meguro, Monira Pervin, Hidefumi Kitazawa, Ai Yoto, Mayu Ishino, Yuki Shimba, Yusuke Mochizuki, Shinji Miura, Ichiro Tokimitsu, Keiko Unno

**Affiliations:** 1 Biological Science Research, Kao Corporation, Akabane, Ichikai-machi, Haga-gun, Tochigi 321-3497, Japan; oonishi.shintarou@kao.com (S.O.); kitazawa.hidefumi@kao.com (H.K.); 2 Tea Science center, University of Shizuoka, Yada, Suruga-ku, Shizuoka 422-8526, Japan; gp1747@u-shizuoka-ken.ac.jp (M.P.); ai_yoto@hotmail.com (A.Y.); unno@u-shizuoka-ken.ac.jp (K.U.); 3 Laboratory of Nutritional Biochemistry, Graduate School of Nutritional and Environmental Sciences, University of Shizuoka, Yada, Suruga-ku, Shizuoka 422-8526, Japan; s16213@u-shizuoka-ken.ac.jp (M.I.); s16214@u-shizuoka-ken.ac.jp (Y.S.); s16215@u-shizuoka-ken.ac.jp (Y.M.); miura@u-shizuoka-ken.ac.jp (S.M.); 4 Department of Health and Food Science, University of Human Arts and Science, Magome, Iwatsuki-ku, Saitama 339-0077, Japan; ichiro_tokimitsu@human.ac.jp; 5 Department of Neurophysiology, School of Pharmaceutical Sciences, University of Shizuoka, Yada, Suruga-ku, Shizuoka 422-8526, Japan

**Keywords:** aging, green tea extracts, oxidative stress, senescence-accelerated mouse prone-8, synaptic plasticity

## Abstract

Unhealthy diet promotes progression of metabolic disorders and brain dysfunction with aging. Green tea extracts (GTEs) have various beneficial effects and alleviate metabolic disorders. GTEs have neuroprotective effects in rodent models, but their effects against brain dysfunction in models of aging fed unhealthy diets are still unclear. Here, we showed that GTEs attenuate high-fat (HF) diet-induced brain dysfunction in senescence-accelerated mouse prone-8 (SAMP8), a murine model of senescence. SAMP8 mice were fed a control diet, HF diet, or HF diet with 0.5% GTEs (HFGT) for four months. The HF diet reduced memory retention and induced amyloid β_1–42_ accumulation, whereas GTEs attenuated these changes. In HF diet-fed mice, lipid oxidative stress, assessed by malondialdehyde levels, was increased. The levels of proteins that promote synaptic plasticity, such as brain-derived neurotrophic factor (BDNF) and postsynaptic density protein 95 (PSD95), were reduced. These alterations related to brain dysfunction were not observed in HFGT diet-fed mice. Overall, our data suggest that GTEs intake might attenuate brain dysfunction in HF diet-fed SAMP8 mice by protecting synaptic plasticity as well as via anti-oxidative effects. In conclusion, GTEs might ameliorate unhealthy diet-induced brain dysfunction that develops with aging.

## 1. Introduction

Alzheimer’s Disease International reported that dementia has become a worldwide health problem with global increases in population sizes and life expectancy [1]. According to their new report, 50 million people live with dementia, and the global social cost reached US$1 trillion in 2018 [2]. Although dementia cannot be cured even with modern drugs and technologies, prevention and early intervention has been widely accepted to reduce risk factors for dementia such as diabetes, hypertension, underactivity, and smoking [3,4].

Unhealthy diet including excessive fats can lead to type 2 diabetes, insulin resistance, hypertension, and other conditions linked to obesity [5,6]. Type 2 diabetes is a risk factor for cognitive impairment [7,8], and insulin resistance is associated with brain atrophy and poor cognitive function in late middle-aged adults [9]. Senescence-resistant inbred strain 1 (SAMR1) and senescence-accelerated mouse (SAM) prone-8 (SAMP8) are regarded as appropriate mouse models for brain dysfunction and non-transgenic models of aging [10,11,12]. High-fat (HF) diet-induced metabolic disorder causes cognitive impairment in SAMP8 mice [12]. The above studies suggest that the induction of metabolic disorders by unhealthy diet, including excessive fats, might induce cognitive impairment with aging.

Green tea extracts (GTEs), which contain high concentrations of polyphenols such as epigallocatechin gallate (EGCg), have been extensively evaluated for their effects on obesity, type 2 diabetes, insulin resistance, and hyperglycemia in humans and rodents [13,14,15,16,17,18,19,20]. The mechanisms responsible for the effects of GTEs on obesity-related diseases may rely on increasing glucose and lipid oxidation [17,21,22,23,24], anti-oxidative stress [13,14], and anti-inflammatory effects [14,15]. In addition, EGCg has a neuroprotective effect [25,26], and GTEs and EGCg improve cognitive function in several rodent models of cognitive dysfunction [10,11,27]. In these studies, EGCg decreased amyloid β (Aβ) accumulation by upregulating a degradation enzyme, neprilysin (NEP; also known as neutral endopeptidase-24.11 or Aβ-degrading protein [28,29]), or improved synaptic function. However, the effects of GTEs against HF diet-induced brain dysfunction are still unclear. We hypothesized that GTEs intake would improve brain dysfunction in HF diet-fed SAMP8 mice, which are considered a useful model for testing the therapeutic potential of food composition against brain dysfunction in diabetes [12].

In this study, we examined changes in brain function (memory retention, brain weight, and Aβ_1–42_ accumulation) during aging in SAMR1 and SAMP8 mice and the negative influences of HF diet on brain function. Then, we investigated the effect of GTEs intake on HF diet-induced changes in the brain of SAMP8 mice. We found that an increase in lipid oxidative stress and deficits in the levels of several proteins are the likely mechanisms of HF diet-induced changes in brain function and the effects of GTEs.

## 2. Materials and Methods

### 2.1. Ethics Statement

The research was approved by the Institutional Animal Care and Use Committee of the University of Shizuoka (approval number: 136068) and conducted in accordance with the National Institutes of Health Guide for the Care and Use of Laboratory Animals and our institutional guidelines. All surgery was performed under anesthesia using 2.5% isoflurane (Wako Pure Chemical Industries, Ltd., Osaka, Japan), and all efforts were made to minimize suffering.

### 2.2. Animals and Diet Preparation

The animal experiments described in this study were carried out simultaneously with those described in our recent publication [19]. In brief, male SAMR1 (Number of samples, *N* = 24) and SAMP8 (*N* = 56) mice were obtained from Japan SLC, Inc. (Hamamatsu, Japan). Mice (5 or 6 per cage) were housed under conventional conditions with a 12-h light/dark cycle and free access to diet and water. During the entire experiment, SAMR1 mice were fed a control (Cont) diet. SAMP8 mice with similar body weights were allocated to three diet groups: Cont (*N* = 24), HF (*N* = 16), and HF with GTEs (HFGT, *N* = 16). Composition of the diets is shown in Table 1. Each diet was prepared using an in-house procedure. A premix of cellulose, vitamins, and minerals with or without GTEs was added to a premix of potato starch, sucrose, and casein. Then, melted lard and corn oil were gradually added while mixing sufficiently to maintain a uniform powder. The diets were divided into portions, dispensed into light-shielding bags, purged with nitrogen to prevent oxidation, sealed and stored at 4 °C until use. The diets were fed for 4 months from 2 to 6 months of age. Diet intake was measured three times per week during the experimental period. Calorie-based average daily amount of food intake per mouse (kcal/mouse/day) did not differ significantly among the groups and was 17.00 (SAMR1; Cont), 17.06 (SAMP8; Cont), 19.06 (SAMP8; HF), and 18.13 (SAMP8; HFGT). Although no significant difference in body weight was observed among any groups of mice at 6 months of age, the HF group of SAMP8 mice had insulin resistance, as assessed by homeostasis model assessment of insulin resistance (HOMA-IR) [19].

Mice at 2 months of age (SAMR1-Cont and SAMP8-Cont; *N* = 8 per group) and at 6 months of age (SAMR1-Cont and SAMP8-Cont, -HF, and -HFGT; *N* = 16 per group) were fasted for 5 h and anesthetized with 2.5% isoflurane. Anesthesia was confirmed by the loss of the pedal reflex, blood samples were collected from the abdominal vena cava under laparotomy, and the mice were exsanguinated. Brains were immediately dissected, weighed, and the cerebral cortex and hippocampus were stored at −80 °C until subsequent analyses.

### 2.3. Catechins Composition and Caffeine Content

GTEs were purchased from Mitsui Norin Co., Ltd. (Tokyo, Japan). The composition of catechins was determined by high-performance liquid chromatography [21]. Catechins content of GTEs and HFGT diets are shown in Table 2.

In the HFGT group, average daily intake of food per mouse was 3.49 g; the calculated average daily intake of total catechins was 13.5 mg per mouse (373 mg/kg-body weight) and that of EGCg was 9.70 mg per mouse (269 mg/kg-body weight). This EGCg dose (0.278% supplementation for 4 months) was similar to those reported to ameliorate learning and memory impairment in aging SAMP8 mice (water supplemented with 0.071% EGCg for 6 months [11]) and to ameliorate metabolic disorders in aging SAMP8 mice (0.32 % EGCg-supplemented diet for 3 months [20]) and in HF-fed C57BL/6J mice (0.32% EGCg-supplemented diet for 4 months [15]).

### 2.4. Memory Acquisition and Retention Test

At 6 months of age, mice were subjected to a step-through passive avoidance test according to Unno et al. [30,31] with minor modifications in the strength of memory stimulation and statistical analysis. In brief, a mouse received an electric foot shock at 80 μA for 1 s when it moved from the light side to the dark side of the chamber. The next day, we examined whether the mice would remain in the light for 300 s. Shorter remaining time in light chamber meant lower memory retention. The data were separated into three time sections (100 s each) in consideration of non-normal distribution of time counts, and analyzed by chi-squared test.

### 2.5. Enzyme-Linked Immunosorbent Assay

The level of Aβ_1–42_ in cerebral cortex was measured by enzyme-linked immunosorbent assay (ELISA) using a human/rat β amyloid (42) ELISA kit Wako, High-Sensitive (Wako Pure Chemical Industries, Ltd.). Samples were prepared as described by Hosoda et al. [32] and Borchelt et al. [33] with minor modifications. Approximately 100 mg of cerebral cortex was homogenized with a Physcotron homogenizer (Microtec Co., Ltd., Tokyo, Japan) in 0.7 mL of 70% formic acid and centrifuged at 100,000× *g* for 1 h to obtain soluble and insoluble fractions. The supernatant was neutralized by diluting it 20-fold in 1 M Tris base. The levels of soluble and insoluble Aβ_1–42_ were measured according to the manufacturer’s instructions. To assess oxidative stress, the level of malondialdehyde (MDA) was measured by thiobarbituric acid reactive species (TBARS) assay using an MDA ELISA kit (Japan Institute for the Control of Aging, NIKKEN SEIL Co., Ltd., Shizuoka, Japan). Approximately 100 mg of cerebral cortex was homogenized with a Physcotron homogenizer (Microtec Co., Ltd.) in 250 µL of RIPA buffer (Wako Pure Chemical Industries, Ltd.). Homogenates were centrifuged at 1600× *g* for 10 min, and the supernatants were used for the TBARS assay according to the manufacturer’s procedure.

### 2.6. Western Blot Analysis

Half of the hippocampus was homogenized with a Physcotron homogenizer (Microtec Co., Ltd.) in 200 μL of RIPA buffer (Wako Pure Chemical Industries, Ltd.) containing complete Protease Inhibitor cocktail and PhosSTOP phosphatase inhibitor cocktail (both from Roche Diagnostics). The lysates were centrifuged at 12,000× *g* for 15 min, and the protein concentration in the supernatants was adjusted to 3 mg/mL with 50 mM DL-dithiothreitol (Sigma-Aldrich, St. Louis, MO, USA) in Laemmli Sample Buffer (Bio-Rad Laboratories, Inc., Hercules, CA, USA). Samples (15 μg protein each) were boiled for 5 min, and subjected to SDS-PAGE through a 4% to 15% gradient gel (Bio-Rad Laboratories, Inc.). Western blotting was performed as in our previous report [19]. Proteins were then transferred to polyvinylidene fluoride membranes (Bio-Rad Laboratories, Inc.) and incubated for 3 h with PVDF Blocking Reagent for Can Get Signal (TOYOBO Co., Ltd., Osaka, Japan). The membranes were incubated overnight with primary antibodies diluted 1:1000 in Can Get Signal Solution 1 (TOYOBO Co., Ltd.), followed by anti-rabbit IgG horseradish peroxidase-linked secondary antibody (#7074) (Cell Signaling Technology, Danvers, MA, USA) diluted 1:2000 in Can Get Signal Solution 2 (TOYOBO Co., Ltd.) for 1 h. Signals were detected using the ECL Prime Western Blotting Detection System (GE Healthcare Japan, Tokyo, Japan) and visualized with a luminescence imager (Ez-capture II, ATTO Co., Tokyo, Japan). Primary antibodies against postsynaptic density protein 95 (PSD95; #2450), synaptophysin (#4329), and β-actin (#4967) were purchased from Cell Signaling Technology; against NEP (ab79423) was purchased from Abcam plc, (Cambridge, UK); and against brain-derived neurotrophic factor (BDNF; AB1534SP) was purchased from Chemicon International, Inc. (Temecula, CA, USA).

### 2.7. Statistical Analysis

**Figure 1:**
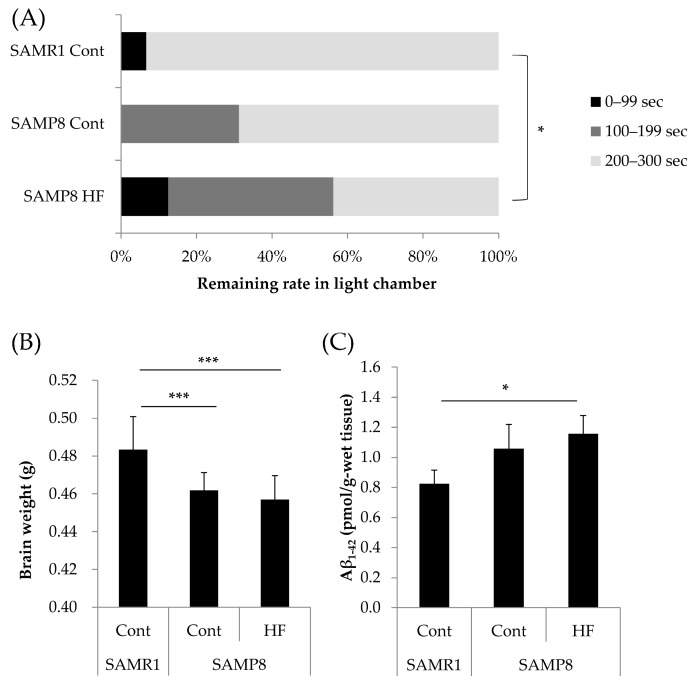
Effects of a HF diet on memory function, total brain weight, and Aβ_1–42_ accumulation in a senescence-accelerated mouse model at six months of age. (**A**) Memory retention was measured by a step-through passive avoidance test one day after the mice acquired memory. (**B**) Total brain weight. (**C**) Aβ_1–42_ accumulation in cerebral cortex. Cont, control diet; HF, high-fat diet. Data are means ± S.D. (16 mice per group). Statistical significance was determined by: (**A**) chi-squared test followed by Kruskal–Wallis one-way analysis of variance and Bonferroni correction; and (**B**,**C**) one-way ANOVA followed by Tukey’s post-hoc test. * *p* < 0.05; *** *p* < 0.001.

**Figure 2:**
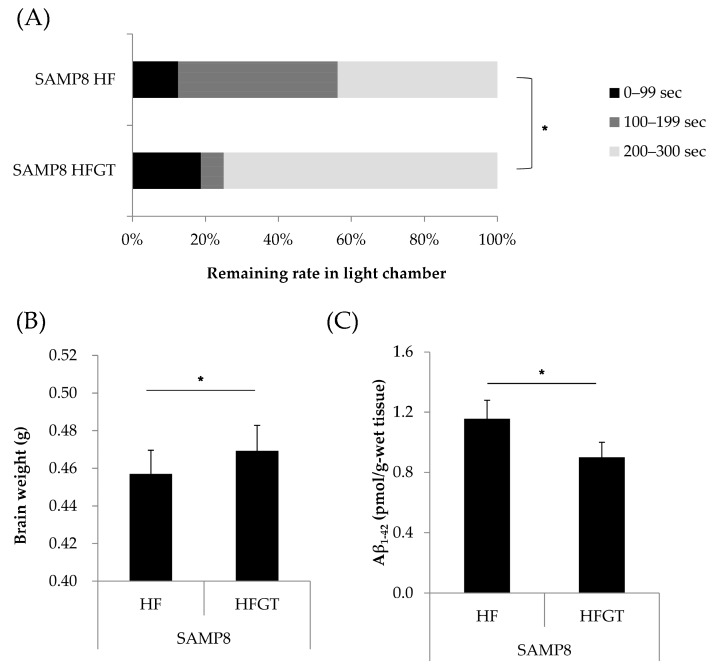
Effects of GTEs intake on memory function, total brain weight, and Aβ_1–42_ accumulation in HF diet-fed SAMP8 mice at six months of age. (**A**) Memory retention was measured as in Figure 1A. (**B**) Total brain weight. (**C**) Aβ_1–42_ accumulation in cerebral cortex. HFGT, HF diet with 0.5% GTEs. Data are means ± S.D. (16 mice per group). Statistical significance was determined by: (**A**) chi-squared test; and (**B**,**C**) Student’s *t*-test. * *p* < 0.05.

**Figure 3:**
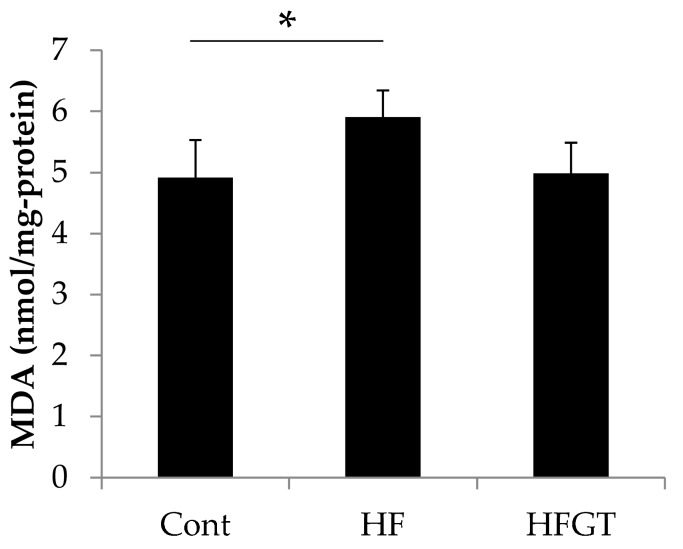
Effects of GTEs intake on HF diet-induced oxidative stress in SAMP8 mice. The levels of malondialdehyde (MDA), a lipid peroxide degradation product, in the cerebral cortex were measured by thiobarbituric acid reactive species (TBARS) assay. Data are means ± S.D. One-way ANOVA followed by Dunnett’s test was used for comparison among groups (Number of samples, *N* = 4). * *p* < 0.05.

**Figure 4:**
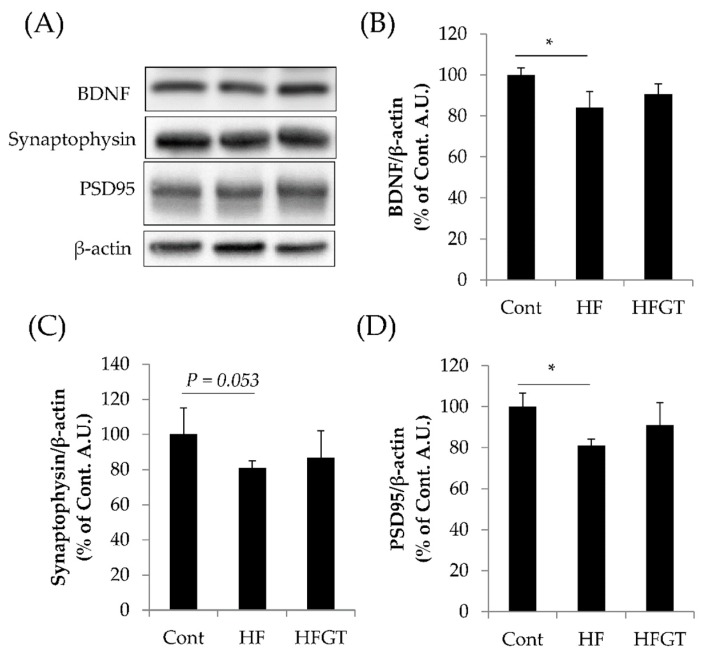
Effects of GTEs intake on the levels of BDNF, synaptophysin, and PSD95 in SAMP8 mice. (**A**) Representative Western blot images (all images are provided in Figure S2); and quantification of protein levels for: (**B**) brain-derived neurotrophic factor (BDNF); (**C**) synaptophysin; and (**D**) postsynaptic density protein 95 (PSD95). Data are means ± S.D. One-way ANOVA followed by Dunnett’s test was used for comparison among groups (*N* = 4). * *p* < 0.05.

Data are expressed as means ± S.D. All data were analyzed using IBM SPSS Statistics Version 24 (IBM Corp., Armonk, NY, USA). The statistical significance of differences in memory function (Figure 1A and Figure 2A) was determined by chi-squared test. In Figure 1A, chi-squared test was followed by Kruskal–Wallis one-way analysis of variance with Bonferroni correction. One-way ANOVA followed by Tukey’s post-hoc test was used for the results presented in Figure 1B,C. Student’s *t*-test was used in Figure 2B,C. One-way ANOVA followed by Dunnett’s test was used in Figure 3 and Figure 4. Values of *p* < 0.05 were considered significant. 

## 3. Results

### 3.1. Effects of a HF Diet on Memory Function, Total Brain Weight, and Aβ_1–42_ Accumulation in a Senescence-Accelerated Mouse Model at Six Months of Age

In comparison with SAMR1 mice, SAMP8 mice fed Cont diet tended to have lower memory retention and SAMP8 mice fed HF diet had significantly lower memory retention (χ^2^ (1) = 10.9, *p* = 0.015). Regardless of the diet, total brain weight was significantly lower in SAMP8 mice than in SAMR1 mice (Figure 1B), which was considered to be represented the phenotype of SAMP8 mice as aging model. The levels of Aβ_1–42_ were significantly higher in SAMP8 mice fed the HF diet than in SAMR1 mice (Figure 1C). None of the above differences were observed between SAMR1 and SAMP8 mice at two months of age, before feeding different diets (Figure S1). The above results suggest that memory dysfunction, brain weight loss and Aβ_1–42_ accumulation were induced by HF diet in SAMP8 mice. 

### 3.2. Effects of GTEs Intake on Memory Function, Total Brain Weight, and Aβ_1–42_ Accumulation in HF Diet-Fed SAMP8 Mice at Six Months of Age

The effects of GTEs on the model of brain dysfunction, which had been confirmed to be induced in SAMP8 mice fed the HF diet, were investigated. Memory retention was significantly higher (χ^2^ (1) = 6.02, *p* = 0.049) in the HFGT than in the HF group (Figure 2A). Total brain weight was significantly increased (Figure 2B) and the amount of Aβ_1–42_ was significantly decreased (Figure 2C) in the HFGT in comparison with the HF group.

### 3.3. Effects of GTEs Intake on HF Diet-Induced Oxidative Stress

To assess oxidative stress, we next investigated the levels of MDA, a lipid peroxide degradation product, in the cerebral cortex (Figure 3). The MDA level was significantly higher in the HF group (5.91 nmol/mg-protein) than in the Cont group (4.91 nmol/mg-protein). GTEs intake decreased the MDA level to 4.99 nmol/mg-protein, i.e., almost to the level in the Cont group.

### 3.4. Effects of GTEs Intake on the Levels of BDNF, Synaptophysin, and PSD95

The levels of BDNF and PSD95 were significantly lower and that of synaptophysin tended to be lower in the HF than in the Cont group (Figure 4). On the other hand, there were no significant differences between the HFGT and Cont groups. These protein levels in the hippocampus might have affected memory acquisition and retention, under the possibility that GTEs attenuated the negative influence on hippocampus by HF diet.

## 4. Discussion

The goal of this study was to assess the effects of GTEs on HF diet-induced brain dysfunction in aging mice. We previously reported signs of diabetes (e.g., insulin resistance as assessed by HOMA-IR) in SAMP8 mice fed 51.7 Kcal%-HF diet for four months [19] and animal experiment in the present work was simultaneously carried out with the above study. Mehla et al. showed that SAMP8 mice had a sign of diabetes (assessed by glucose tolerance test) and brain dysfunction after three months of 60 Kcal%-HF feeding [12]. Although our experimental design was not identical to that of Mehla et al., our experiments reproduced their results regarding brain dysfunction in SAMP8 mice with HF diet. Our new findings reported here indicate that GTEs intake attenuates brain dysfunction in SAMP8 mice with HF diet.

Deficits in the brain function of SAMP8 mice are believed to increase lipid oxidative stress in cerebral cortex [34]. Oxidative stress is one of the factors that induce Aβ_1–42_ production [35]. Aβ_1–42_ accumulation and MDA elevation, and NEP decline (Figure S3; NEP levels tend to be decreased) in the HF group suggest that lipid oxidative stress under excessive fat intake was related to Aβ_1–42_ accumulation caused by a decline in the Aβ_1–42_ degradation enzyme NEP. These changes in oxidative stress and NEP levels were not observed in the HFGT group. Although several studies show that oxidative stress induces Alzheimer’s disease progression with Aβ accumulation [36,37], GTEs could prevent oxidative stress in the elderly population [38] and in aging or disease rodent models [26,39,40,41,42,43]. In addition, NEP expression in rodent model is upregulated by intragastric administration of EGCg (5–5 mg/kg body weight) for 60 days [10]. Therefore, GTEs intake might alleviate Aβ_1–42_ accumulation by decreasing oxidative stress and possibly upregulating NEP in the HF diet fed SAMP8 mice.

Memory retention is affected by a variety of factors, such as Aβ accumulation and oxidative stress [11,12], and by synaptic plasticity; it requires a variety of proteins such as BDNF, synaptophysin, and PSD95 [11,44,45,46,47,48,49]. BDNF plays important roles in learning and memory by regulating synaptic plasticity [44,45]. When memory is acquired, presynaptic and postsynaptic regions communicate with each other. Synaptophysin, a marker of the presynaptic region and synaptic vesicles, is required for synaptic vesicle transmission [46,47]. PSD95, a marker of the postsynaptic region, is required for synapse formation and reception of synaptic stimuli [47,48,49]. Li et al. reported that drinking 0.05–0.10% GTEs with normal diet for six months increased BDNF and PSD95 levels in 4–10-month-old SAMP8 mice [11], and drinking 0.05% GTEs with normal diet for six months increased PSD95 levels in 14–20-month-old C57BL/6J mice [39]. Similarly, our experiments showed that GTEs intake decreased the extent of BDNF and PSD95 reduction by HF diet in SAMP8 mice. EGCg protects neurons from sevoflurane-induced apoptosis via BDNF signaling [50]. Overall, the above data suggest that GTEs protect against HF diet-induced neurological deficits that affect synaptic plasticity.

Our study has the following limitations. GTEs were used as a crude mixture that contained mainly catechins such as EGCg, and the exact composition of GTEs was not determined; the composition can be affected by the extraction method [51,52]. The effective compound and its effective dose were not determined. Only a single dose of GTEs (0.5% of the diet) was tested, and the most effective dose of GTEs remains to be determined; it may be lower or higher than the concentration used. Overall, comparative analysis of several types and doses of GTEs and purified catechins is needed. A limitation in the experimental design is that we used only male mice to avoid the influence of menstrual cycles on behavior or metabolism. Because sex differences in cognitive function during aging are documented in humans and mouse models of AD [53,54], a similar study on female mice is needed to further clarify the deficit of brain function in aging induced by HF diet and the counter effect of GTEs.

No effective drugs are currently available on the market for the prevention or early intervention for dementia [55]. As an alternative to developing drugs, food constituents have also been investigated in intervention studies [25,56,57,58,59,60,61,62]. To date, there have only been two intervention studies of GTEs [25,56]; both cited studies investigate the effect of acute intervention to brain function with single administration in healthy adults. On the other hand, long-term consumption of GTEs has intervention effects on obesity-related diseases [13,14,16,17], which are considered risk factors for dementia [3,4,5,6,7,8,9]. Our findings show that four-month GTE intake attenuates brain dysfunction in HF diet-fed SAMP8 mice; this effect is accompanied by a decrease in oxidative stress and Aβ_1–42_ accumulation, which are considered important causative factors in AD pathogenesis and progression [35,63]. Although our finding needs to be validated in humans, our study suggests a way to solve problems related to cognitive dysfunction caused by oxidative stress and Aβ_1–42_ accumulation. Therefore, daily intake of GTEs as functional nutrients might be important in the modern society where the incidence of brain dysfunction, such as dementia and AD, is increasing because of unhealthy diet.

## 5. Conclusions

Our experiments showed that unhealthy diet exacerbated aging-related brain dysfunction with Aβ_1–42_ accumulation accompanied by lipid oxidative stress, whereas the intake of GTEs ameliorated memory retention and decreased Aβ_1–42_ accumulation in the cerebral cortex of HF diet-fed SAMP8 mice. These results suggest that GTEs intake is important for brain function under excessive fat intake during aging.

## Figures and Tables

**Table 1:** Composition of experimental diets fed to mice.

	Cont	HF	HFGT
Lard	0	5	5
Corn oil	5	25	25
Potato starch	66.5	28.5	28
Sucrose ^a^	0	13	13
Casein	20	20	20
Cellulose	4	4	4
Vitamins (AIN-76)	3.5	3.5	3.5
Minerals (AIN-76)	1	1	1
Green tea extracts (GTEs) ^b^	0	0	0.5
Energy ^c^	%		
Protein	20.5	15.7	15.8
Fat	11.3	51.7	51.9
Carbohydrate	68.2	32.6	32.3

Cont, control diet; HF, high-fat diet; HFGT, HF diet with 0.5% GTEs; diet compositions are indicated in % (w/w). ^a^ Obtained from Wako Pure Chemical Industries, Ltd. (Osaka, Japan). ^b^ Obtained from Mitsui Norin Co., Ltd. (Tokyo, Japan). GTEs include 22.4% of unidentified components and minimum amount of caffeine shown in Table 2. ^c^ Percent of kcal of each macronutrient. Other ingredients were obtained from Oriental Yeast Co., Ltd. (Tokyo, Japan).

**Table 2:** Catechins content of GTEs and HFGT diets.

	% of GTEs	g/100 g of GTEs	mg/100 g of HFGT Diet
EGCg, epigallocatechin gallate	71.7	55.6	278
ECg, epicatechin gallate	16.2	12.5	62.6
GCg, gallocatechin gallate	6.25	4.85	24.2
EGC, epigallocatechin	2.76	2.14	10.7
EC, epicatechin	1.24	0.962	4.81
Cg, catechin gallate	0.77	0.597	2.99
GC, gallocatechin	0.65	0.504	2.52
C, catechin	0.24	0.186	0.93
Other catechins	0.26	0.202	1.01
Others *		22.4	
Total	100	100	388

* Others include unidentified components. Catechins purity of GTEs was 77.6%; The caffeine content was 0.151 g/100 g of GTEs.

## Supplementary Materials

The following are available online at https://www.mdpi.com/2072-6643/11/4/821/s1, Figure S1: Memory retention, total brain weight, and Aβ_1–42_ accumulation in SAMR1 and SAMP8 mice at 2 months of age. Figure S2: Synapse-associated protein levels in SAMP8 mice at 6 months of age. Figure S3: Effects of GTEs intake on NEP levels in SAMP8 mice.
