# Supplementary material for: Green Tea Extracts Attenuate Brain Dysfunction in High-Fat-Diet-Fed SAMP8 Mice
Source: Nutrients. 2019 Apr 11;11(4):821. doi: 10.3390/nu11040821 (PMC6521105; doi:10.3390/nu11040821)
Supplement: Supplementary file 1 [file nutrients-11-00821-s001.pdf]

nutrients

MDPI

*Type of the Paper (Article)*

**Title: Green tea extracts attenuate brain dysfunction in high-fat diet–fed SAMP8 mice**

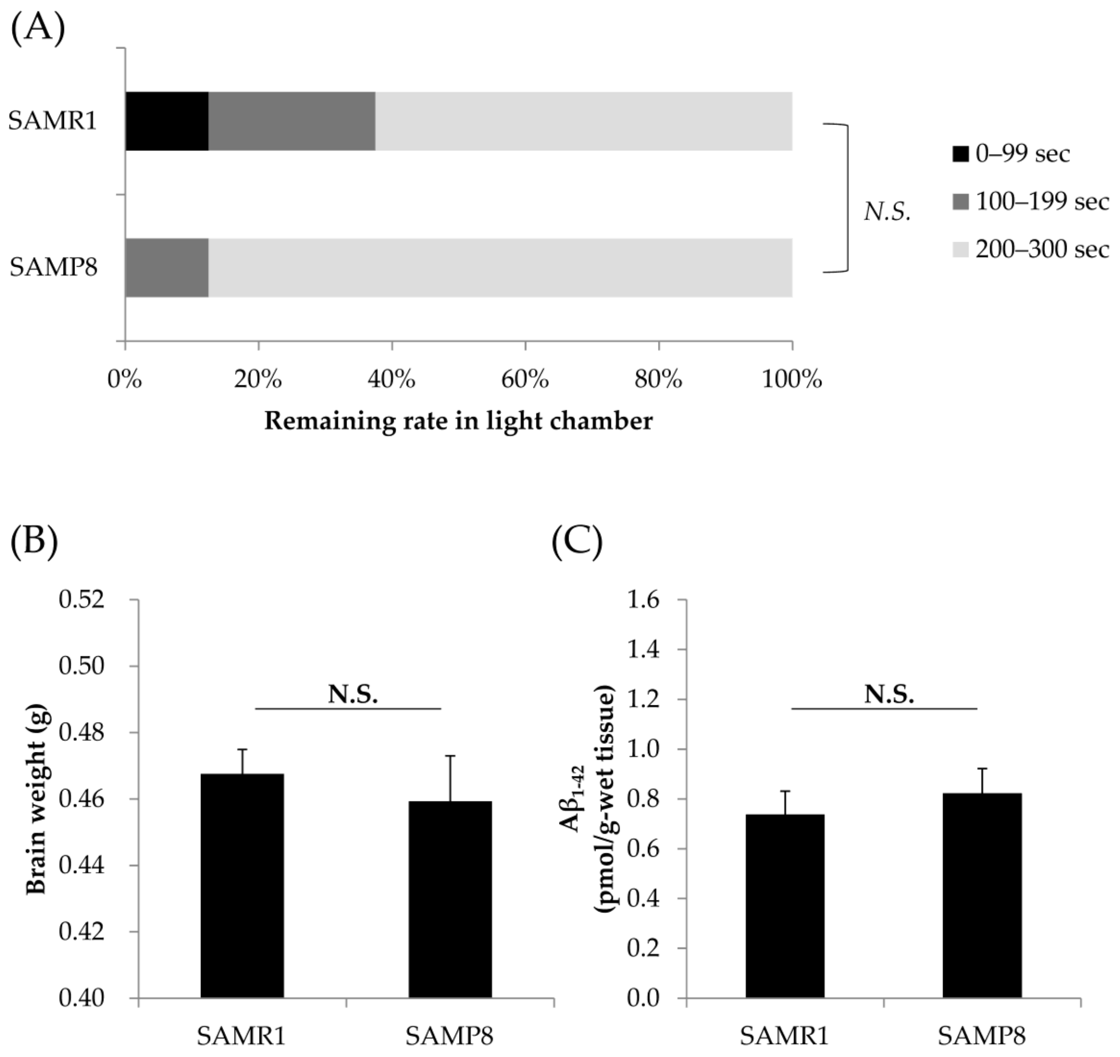


**Figure S1:** Memory retention, total brain weight, and A$\beta_{1\text{-}42}$ accumulation in SAMR1 and SAMP8 mice at 2 months of age. (**A**) Memory retention was measured by a step-through passive avoidance test 1 day after the mice acquired memory at 2 months of age. Memory retention was not different between SAMR1 and SAMP8 mice at 2 months of age; $\chi 2$ (1) = 1.67, *P* = 0.435. (**B**) Total brain weight and (**C**) A$\beta_{1\text{-}42}$ accumulation in cerebral cortex were not different between SAMR1 and SAMP8 mice at 2 months of age. Data are means ± S.D. (8 mice per group). Statistical significance was determined by (**A**) chi-squared test; (**B**,**C**) Student's *t*-test.

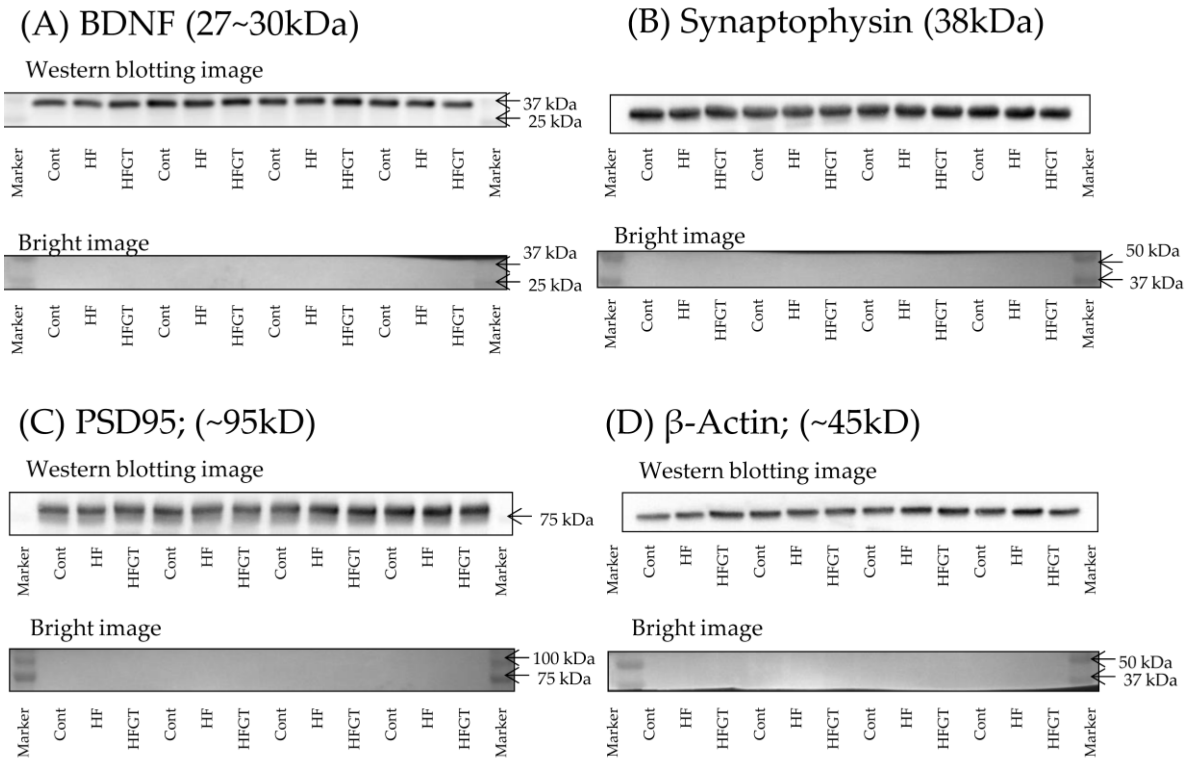


**Figure 2.** Synapse-associated protein levels in SAMP8 mice at 6 months of age. Western blot images were shown for (**A**) BDNF, (**B**) synaprophysin, (**C**) PSD95, and (**D**) β-Actin. BDNF were detected as homodimer form about 27 to 30 kDa band size. Precision Plus Protein™ Dual Color Standards (Bio-Rad Laboratories, Inc., Hercules, CA) were used for molecular markers.

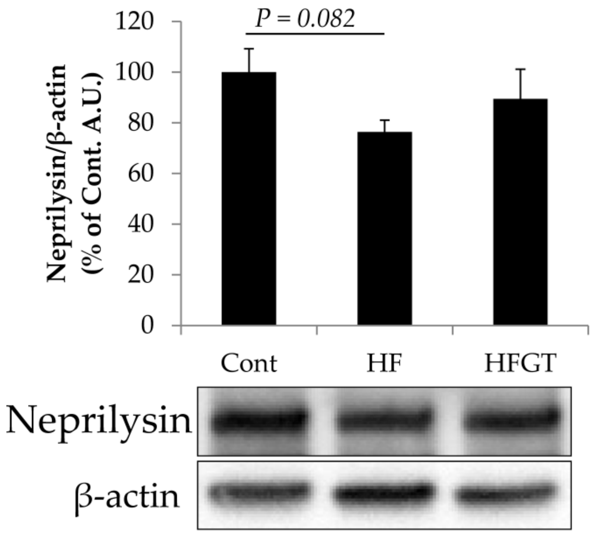


**Figure 3.** Effects of GTEs intake on NEP levels in SAMP8 mice. Quantification of protein levels (upper) and representative western blot images (lower panels) are shown. Western blotting showed that NEP levels tended to be lower by 23.6% in SAMP8 mice fed HF diet than in the Cont group. GTEs intake reduced this difference to 10.5%. The result suggests that GTEs counteracted HF diet–induced $A\beta_{1-42}$ accumulation by increasing the NEP level. Data are means ± S.D. One-way ANOVA followed by Dunnett's test was used for comparison among groups ($N$ = 4).
